# Under the coconut palm – a retrospective analysis of trauma incidents caused by falling coconuts presenting to emergency department at a tertiary care centre in coastal India

**DOI:** 10.1186/s12245-025-00816-4

**Published:** 2025-02-14

**Authors:** A. Sai Deepak, Aaditya Katyal, Neeraja A Nair, Tanvee Walia, Rachana Bhat

**Affiliations:** 1 Department of Emergency Medicine, Kasturba Medical College, Manipal, Manipal Academy of Higher Education (MAHE), Manipal, India; 2 Kasturba Medical College, Manipal, Manipal Academy of Higher Education (MAHE), Manipal, Karnataka India

**Keywords:** Falling coconuts, Coconut fall-related trauma, Coconut falls, Coastal India, Emergency medicine

## Abstract

**Background:**

Injuries due to falling coconuts are a common yet underreported form of trauma in the tropical regions. Although these might appear insignificant at first glance, the physical forces involved are potentially fatal. Despite their global prevalence, research on this subject remains scarce, making it a neglected public health concern. This study seeks to bridge these gaps by analysing the affected demographics, contributing factors and injury patterns. By enhancing the understanding of coconut fall-related injuries, this research seeks to create awareness about dangers of falling coconuts and inform the development of effective public health strategies to mitigate their impact.

**Methods:**

A retrospective study was conducted over a period of 3 years from January 2021 to December 2023 at a tertiary care centre on the southern coast of India. Patients who presented to emergency with coconut-fall related injuries were identified through a comprehensive review of nursing ledgers. Additional data including imaging, consultations & treatment details were retrieved from patient files and electronic medical records. Descriptive statistics of the recorded data like demographic variables, time of injury, injury patterns, injury severity score (ISS) and ED disposition were analysed by using Microsoft Excel 365.

**Results:**

The study population included 17 males and 12 females. Most patients were within the age group of 40–60, which comprised 48% of the total participants. The months of September and October reported the highest frequency of cases. Out of the 29 patients, 14 were farm workers who sustained coconut fall-related injuries. Injury patterns varied from mild soft tissue injuries to severe TBIs which include SDH and SAH. There were 3 patients who required surgery, and 7 patients were admitted. There were no fatalities reported, and average hospital stay was 4.5 days.

**Conclusion:**

Coconut fall-related injuries in tropical regions is a significant but less recognised public health issue. Our study shows the necessity of seasonal preventive strategies, public awareness and safety measures for high-risk population like outdoor workers and older adults. Community focussed interventions, such as regular coconut tree pruning, installation of coconut safety nets and educational campaigns will help to reduce the incidence and severity of these injuries.

**Supplementary Information:**

The online version contains supplementary material available at 10.1186/s12245-025-00816-4.

## Background

Coconut trees (Cocos nucifera), fondly referred to as the “tree of life,” are indispensable to tropical regions, especially Southeast Asia. These iconic trees are deeply intertwined with the economic, cultural, and ecological fabric of the communities they sustain. Offering nourishment through their fruit, versatile materials for construction, and a steady source of income, coconut palms are more than just a symbol of abundance for the local communities—they are lifelines. However, behind this benevolent image lies an inconspicuous yet significant danger: the risk of falling coconuts. Though it may seem trivial at first glance, this hazard has serious consequences for public safety.

Each coconut can weigh up to 4 kg, and when dropped from a height of 30 m or more, the force of impact exceeds an astounding 1 metric ton force [1]. Such an impact is more than enough to cause severe injuries or even fatalities. In heavily populated areas, where people often work or gather under the shade of coconut palms, this danger becomes alarmingly real. Despite the importance of coconut trees to local livelihoods, their unintended risks pose a public health challenge that warrants urgent attention.

The hazards posed by falling coconuts were first highlighted in 1984 by Barss, who studied theses dangers in Papua New Guinea. His findings illustrated that over a period of four years, coconut fall-related injuries constituted 2.5% of trauma cases at a local hospital. Most of these injuries were head traumas, some requiring advanced surgical interventions like craniotomies. Notably, fatalities were also recorded, shedding light on the serious and sometimes fatal consequences of what might appear to be a benign hazard. Barss’ work was instrumental in identifying coconut falls as an overlooked yet critical public health concern in tropical regions [1].

Falling coconuts are not the only hazard associated with these trees. In rural Melanesian communities, for example, Barss and colleagues documented another significant risk: injuries sustained while climbing coconut trees. This traditional practice, often carried out by young men to harvest coconuts, frequently resulted in falls. These occupational hazards emphasize how dependence on coconut trees for subsistence can come at a steep physical cost to those involved in their upkeep [2].

Building on this early research, Mulford and colleagues conducted a cross-sectional study in 2001 in the Solomon Islands focusing on coconut palm-related injuries. The authors found out that these injuries were common across the Pacific Islands’ regions, often leading to debilitating outcomes. Deaths and injuries because of falls from coconut trees or from percussion by coconuts were prevalent especially among young with more than 80% of such cases being 6–20 years of age, working or residing around the palms [3].

Children are particularly vulnerable to serious injuries, a fact again highlighted in a three-year clinical audit conducted by Rehan et al. in the Solomon Islands which revealed that children constituted a significant proportion of patients hospitalized for coconut tree-related trauma. Almost one-third (31%) of the injured children needed referral to a regional hospital, suggesting that injuries in these admissions were severe enough to exceed the capabilities of the local healthcare facility. Rehan and colleagues emphasized the need for targeted community health initiatives to protect children and reduce the incidence of such injuries [4].

Case reports further illustrate the gravity of injuries caused by falling coconuts. In one instance from India, a patient suffered isolated internuclear ophthalmoplegia, a rare neurological condition, after being struck by a falling coconut [5]. In another case from Indonesia, a patient was diagnosed with corpus callosum hemorrhage, highlighting the devastating potential of coconut falls to cause severe traumatic brain injuries [6]. These cases serve as sobering reminders of how an everyday occurrence in tropical regions can lead to complex, life-threatening medical conditions.

What makes the situation even more pressing is the underreporting and lack of awareness surrounding these injuries. Unlike other public health issues that receive widespread attention, coconut-related trauma is often overlooked, dismissed as an unlikely or insignificant risk. However, the data paints a different picture. Whether it is the hospitalization of children, the occupational hazards faced by coconut harvesters, or the grave injuries documented in case reports, the evidence highlights a clear need for interventions to mitigate this unique public health challenge.

Given the relatively high burden of patients presenting with injuries due to falling coconuts observed at our tertiary care centre in coastal India, this study was conceived to systematically examine the patterns, causes, and impact of these injuries. Our goal is to provide a coherent understanding of the population affected and to propose evidence-based measures to reduce the risk of trauma caused by falling coconuts.

## Methods

This was a retrospective study carried out over 3 years between January 2021 to December 2023 at a tertiary care centre located at the southern coast of India. The purpose of the study was to investigate demographic features, seasonal variation, injury patterns, and the outcomes of patients reporting with injuries sustained from falling coconuts. An approval from the Institutional Ethics Committee (approval number IEC1: 187/2024) was sought prior to the commencement of the study to assure compliance with ethics throughout the entire research process.

### Study population

The study population investigated included the patients who attended the emergency department with the history of injuries attributable to falling coconuts during the period of the study from January 2021 to December 2023. Case identification was performed by screening the medical records obtained from the hospital. Inclusion criteria encompassed all patients with known injury because of coconut falls, male or female, without any age restrictions. Exclusion criteria eliminated cases where there were no coconut fall-related injuries or where details relating to the injury of the patient were missing or deemed inadequate.

### Data collection

In the emergency department, patient presentations were documented in a handwritten ledger maintained by the nurses conducting initial assessments for all acute cases. Each patient’s record was entered on a single line, capturing details such as the patient demographics with unique hospital ID, presenting complaints, diagnosis, consultations sought, duration of ED stay and disposition. Each ledger spanned three months of data. For this study, twelve ledgers were reviewed to identify emergency presentations recorded as coconut fall-related trauma out of all patients documented under the heading “occupational injury” as per hospital policy. Patients identified through the ledger review were searched in electronic hospital records and were further cross-checked with the IDs obtained from the hospital medical records department; thus, all patients with a coconut fall-related injury were reliably extracted from the database. This information was further supplemented with a thorough manual review of corresponding patient files obtained from medical records department.

Once identified, the files attributed to coconut tree-related trauma were cross-checked twice by a team of residents to ensure accuracy before extracting information and recording it in the Excel database. In order to gather all the relevant data from the records in an organized manner, a comprehensive proforma was developed to include key data points like demographic details, time & month of incident, mechanism of injury – type and severity with an emphasis on specific body regions & Injury Severity Score (ISS), length of hospital stay, treatments received as well as the ED disposition – whether the patient was discharged, left against medical advice or admitted.

### Data analysis

**Fig. 1:**
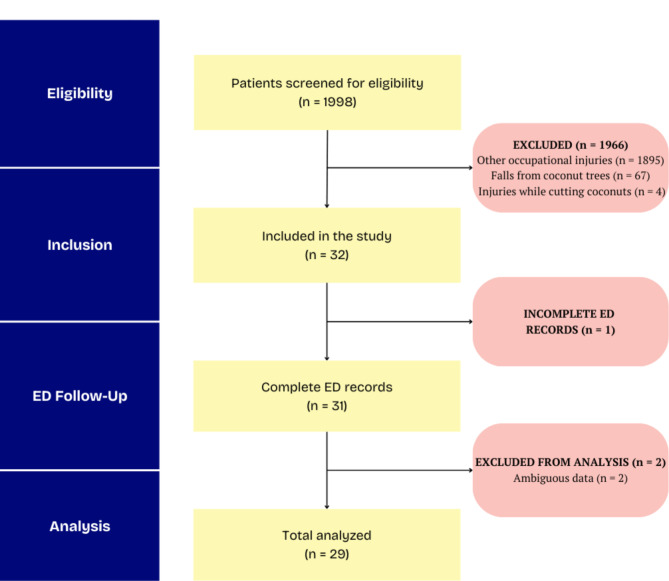
STROBE flow diagram

Data analysis was performed by using Microsoft Excel 365. Categorical variables including age groups, gender, time of injury, seasons, injury patterns and ED disposition were analysed as frequency and percentages. Descriptive statistics were also calculated for the continuous variables such as injury severity scores. A STROBE flow diagram is presented to illustrate the step-by-step process of patient inclusion in the study (Fig. 1).

## Results

**Table 1:** Demographic variables

Demographic variable	Values
**Gender**	
Male	17 [58.7%]
Female	12 [41.3%]
**Age group**	
0–18 years	2 [6.8%]
18–40 years	4 [14%]
40–60 years	14 [48.3%]
60–80 years	9 [31%]
**Time of incident**	
Morning (6 am – 12 pm)	16 [55.3%]
Afternoon (12 pm – 4 pm)	5 [17.2%]
Evening & Night (4 pm – 6 am)	8 [27.5%]
**Month of incident**	
January – February	6 [20.6%]
March – April	4 [13.7%]
May – June	4 [13.7%]
July – August	2 [6.8%]
September – October	9 [31%]
November – December	4 [13.7%]
**Occupation**	
Farm workers	14 [48.3%]
Tree Climbers	10 [34.5%]
Others	5 [17.2%]

There were 29 individuals who reported injuries due to falling coconuts during the 3-year study period. Of the 29 documented cases, 17 were males while 12 were females, and most of them were aged between 40 and 60 years (48.3%). Another 31% were aged between 60 and 80 years and there were only 2 reported cases involving children. The injuries largely occurred during the daytime especially during morning hours – 6 am to 12 pm (Table 1).

### Seasonal and demographic patterns

With regards to the seasons, most coconut injuries occurred during the post-monsoon season between September and October owing to nine recorded cases, highlighting seasonal pattern in coconut fall-related injuries. The activities undertaken by most patients were outdoors, under or around the coconut trees explaining the relationship between the proximity of activities and risk of suffering injuries (Table 1).

### Occupational distribution

Out of the 29 patients, 14 (48.3%) were farm workers who sustained coconut fall-related injuries. Tree climbers accounted for the next prone subgroup comprising of 10 cases (34.5%). A smaller subgroup (Others − 17.2%) comprised of young children, women and elderly who were affected by coconut fall-related injuries (Table 1).

### Injury profiles

**Table 2:** Injury profile of patients

Injuries	Number of patients affected
**Type of Injury**	
Soft tissue injury	8 [27.5%]
Head and neck injury	6 [20.6%]
Thoracic injury ¬ Pneumothorax (3) ¬ Haemothorax (1)	4 [13.7%]
Facial injury	5 [17.2%]
Skeletal injury ¬ Radius fracture (3) ¬ Proximal Interphalangeal joint fracture (1)	4 [13.7%]
No injury	2 [6.8%]
**Sub-classification of fractures**	
Skull bones	3
Long bones	3
Facial bone	3
Axial skeleton ¬ Rib fracture (3) ¬ Clavicle fracture (1)	4

**Fig. 2:**
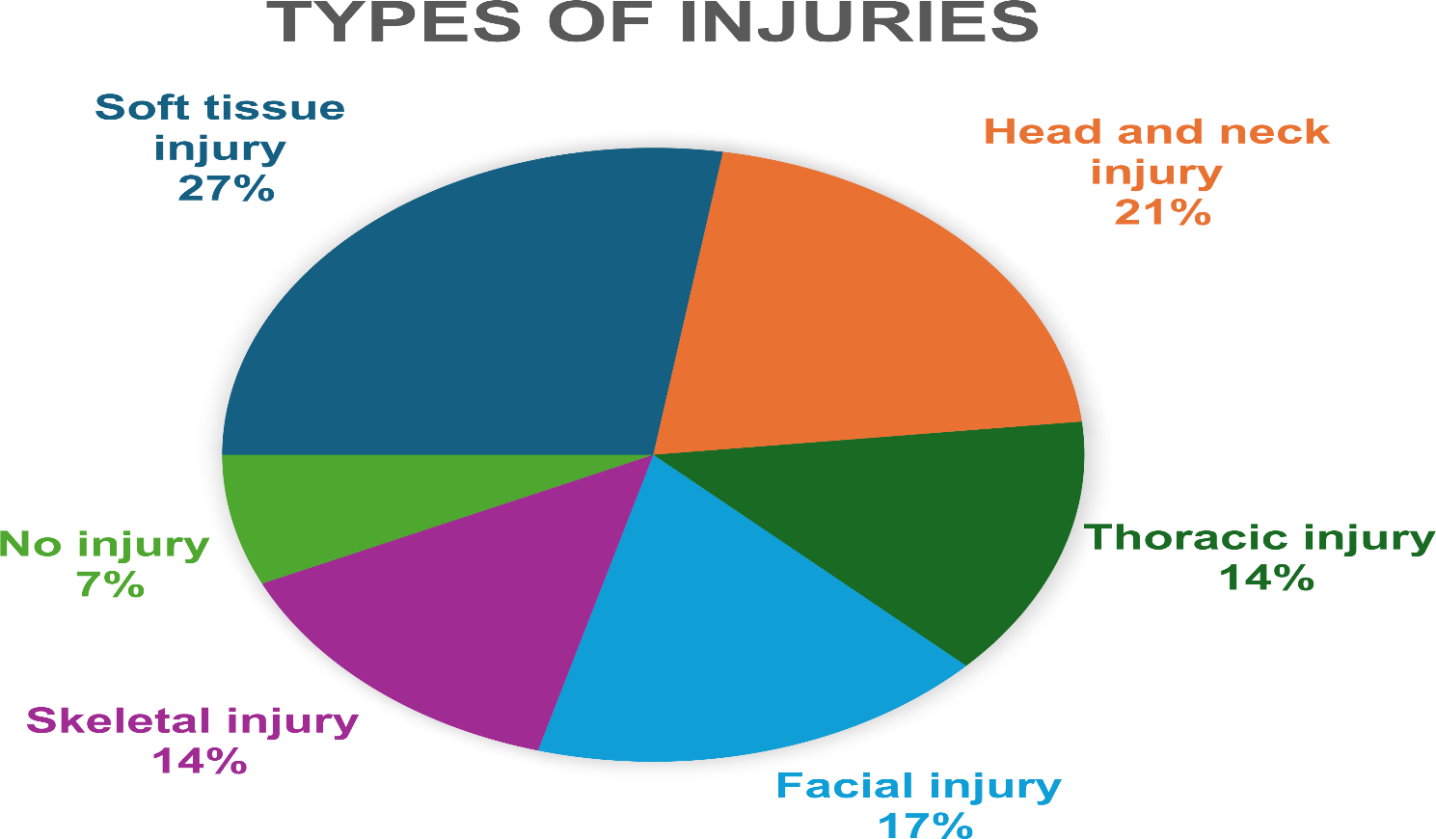
Injury profile of patients

The injury profile showed that there were different types of injuries sustained, the most common being soft tissue injuries mostly affecting head and neck region. Most of these injuries were superficial and treated on an outpatient basis. However, seven patients required hospital admission for more complicated trauma that included different types of traumatic brain injury,

- Subdural Hematoma (SDH) with midline shift was observed in one patient.
- Subarachnoid Haemorrhage (SAH) was present in another patient.
- Three patients suffered cerebral contusions, necessitating monitoring in the intensive care unit (ICU).

Three additional patients sustained significant maxillofacial injuries, such as fractures in the dentoalveolar and nasal bones, along with temporomandibular joint (TMJ) dislocation. 4 patients presented with history of fall of coconut over the thoracic region leading into development of pneumothorax, requiring intercostal drainage (ICD) insertion, while another patient presented with hemothorax and multiple rib fractures, which also required ICD insertion. Five patients sustained extremity fractures; of these, three cases involved distal radius fractures, 2 cases were managed with splinting and one with surgical intervention. Notably, two patients exhibited no visible external injuries despite reported impact from coconut falls (Table 2; Fig. 2).

### Hospitalization and ED disposition

**Fig. 3:**
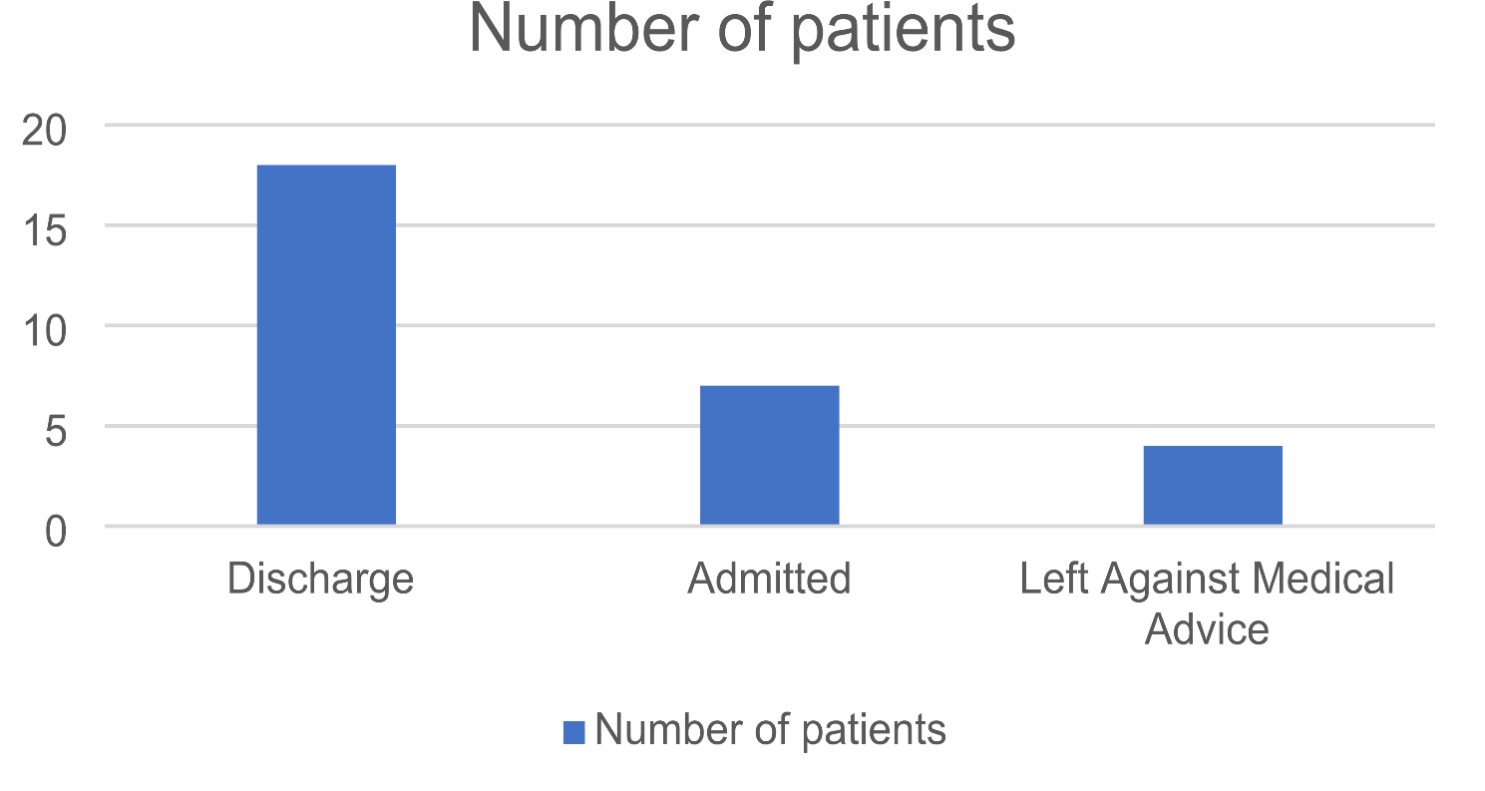
ED disposition

Among 29 patients presented, 18 patients were discharged, 7 required admission and 4 left against medical advice (Fig. 3).

Among the patients requiring admission, the average hospital stay was 4.5 days, with a mean of 1 day spent in the ICU by the patients requiring surgery. Surgical interventions were necessary for three patients:

1. One patient underwent burr hole evacuation for SDH.
2. Another required surgical elevation of a depressed skull fracture.
3. Open reduction and internal fixation was performed on a patient with a distal radius fracture.

None of the admitted patients required blood transfusions, and no mortality was observed at the 28-day follow-up. All admitted patients were discharged in stable condition.

### Injury severity and ISS scoring

**Fig. 4:**
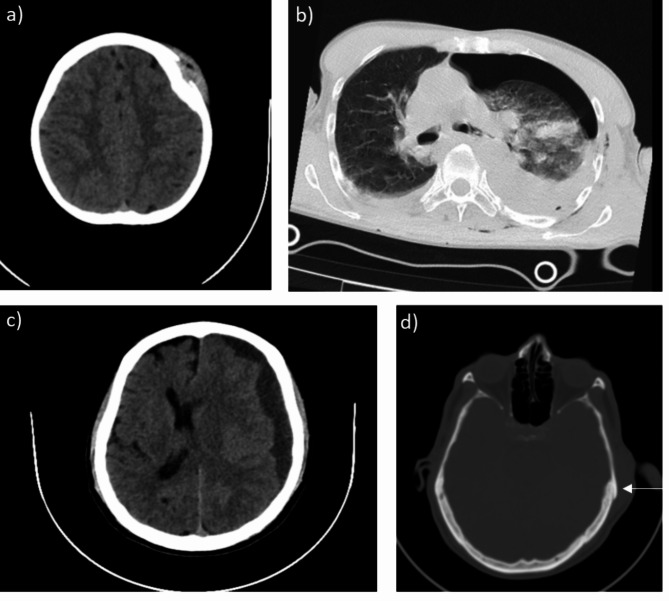
**a**) plain CT showing depressed frontal skull fracture **b**) plain CT showing left sided hemopneumothorax **c**) plain CT showing SDH with midline shift **d**) Plain CT showing undisplaced left temporal bone fracture (arrow)

The Injury Severity Score (ISS) system was used to assess severity of the injuries:

- Mild injuries were the most common, primarily comprising soft tissue injuries that did not necessitate admission. [*n* = 13, 44.8%]
- Moderate injuries included maxillofacial fractures, extremity fractures and small pneumothorax, managed with either outpatient follow-up or brief hospitalization. [*n* = 8, 27.5%]
- Severe injuries were limited to cases involving intracranial trauma, rib fractures with haemothorax, and other injuries requiring intensive monitoring or surgical interventions (Fig. 4). [*n* = 6, 20.6%]
- ISS score ranged between a maximum of 16 to a minimum score of 0.

The ISS analysis suggested that injury severity increased with age, as older patients had more severe injuries and longer hospital stays.

## Discussion

This retrospective study is first of its kind to systematically assess coconut fall-related injuries. It provides valuable insights into the spectrum of injuries caused by falling coconuts, emphasizing the need for public health awareness and preventive strategies. Our findings demonstrate that coconut fall-related injuries can range from minor soft tissue injuries to life-threatening traumatic brain injuries (TBIs), posing a significant risk in tropical and coastal areas where coconut trees are a central part of the landscape and livelihood.

Most patients in our study were middle-aged adults (40–60 years), with a slight predominance of males (17 out of 29 cases). This aligns with previous research indicating that adult males, particularly who worked around coconut trees, are often at higher risk for coconut tree-related injuries due to occupational exposure. Studies by Mulford et al. [3] in the Pacific Islands and Barss et al. [1] in Papua New Guinea similarly highlight a higher prevalence of injuries among individuals engaged in outdoor activities or living in densely populated areas near coconut palms. Seasonal trends in our study revealed that most injuries occurred during September and October, the post-monsoon season, a period associated with increased risk of falling coconuts due to strong winds and scattered rainfall, supporting findings by Barss et al. [1] and Mulford et al. [3], who also noted heightened injury incidence during seasons with elevated environmental risks.

Farm workers and tree climbers formed the largest groups of affected individuals, accounting for 48.3% and 34.5% of cases, respectively, highlighting the direct and indirect occupational hazards associated with coconut farming. The dangers faced by tree climbers are particularly evident, as emphasized by Barss et al., stating that most coconut harvesters are likely to suffer an injury while climbing tall palms [2]. In addition to occupational risks, the study also identified injuries among children, women, and elderly individuals who were incidentally exposed to falling coconuts, often while performing daily activities near coconut trees.

The injuries recorded ranged from soft tissue injuries to severe traumatic brain injuries (TBIs), particularly in cases where patients were struck on the head. Head injuries, including subdural hematoma (SDH) with midline shift, subarachnoid haemorrhage (SAH), and cerebral contusions, were among the most serious outcomes. This distribution aligns with findings from Escoffery and Shirley, who reported fatal head injuries from coconut and branch falls, specifically noting the risk of skull fractures in cases involving direct impacts on the head [7]. Correspondingly, Barss reported of head trauma caused by coconuts which needed surgical interventions for two patients [1]. These outcomes are consistent with prior studies that have highlighted dangers posed by falling objects from tall trees, such as coconuts & Brazil nuts, emphasizing their potential to cause severe injuries [3, 7, 8]. The immense force generated by a falling coconut, estimated at over 9,800 N when dropped from a height of 30 m, makes head injuries particularly devastating [1]. Using the principle of energy conservation, the impact energy transmitted by a falling coconut to its target is calculated as approximately 1800 J of kinetic energy (KE). The relationship between the KE of the impactor and its diameter is defined by the Blunt Criterion (BC), a model validated in 2009 for predicting skull fractures by Raymond et al. [9]. In this study, a striking force of 9800 N corresponds to approximately 100% probability of sustaining skull fracture based on the injury risk prediction curves.

Thoracic injuries, though less common, were notable in several cases, with patients presenting with pneumothorax, hemothorax, and multiple rib fractures. Skeletal injuries, including fractures of the skull, facial bones, and long bones, further illustrate the diverse and often complex nature of coconut fall-related trauma.

The Injury Severity Score (ISS) analysis revealed that injury severity tended to increase with age, as older adults were more likely to sustain severe trauma requiring hospitalization or intensive care. This finding aligns with broader evidence indicating that elderly patients often experience worse outcomes due to preexisting comorbidities, poor physiological reserves and a slow recovery [3].

Out of the 29 cases included in this study, 7 were hospitalized with an average hospital stay of 4.5 days, with a mean of 1 day spent in the ICU by the patients requiring surgery. Three patients were managed with surgical interventions which included evacuation of clot through burr hole, surgical elevation of depressed skull fracture, and open reduction of fractured distal radius. These interventions are consistent with past studies where patients had to be operated on to relieve raised intracranial pressure and other related complications of head injuries [1, 2, 7]. However, contrary to Barss [1] and Escoffery et al. [7] who reported some deaths, all the patients who were included in our study were hemodynamically stable at discharge. Thoracic trauma patients who developed pneumothorax and hemothorax secondary to coconut fall required ICD insertion adding to the gamut of possible injuries.

The findings of this study highlight the urgent need for targeted public health interventions to mitigate the risks associated with coconut trees. Educational campaigns could play a pivotal role in raising awareness among vulnerable populations, including farm workers, tree climbers, children, and elderly individuals. These campaigns should emphasize the importance of avoiding high-risk areas during windy or rainy seasons and encourage the use of protective gear, such as helmets, for workers engaged in coconut harvesting.

Structural interventions, such as the installation of safety nets around coconut palms, offer a practical solution to reduce injuries in public and residential spaces. Regular tree maintenance, including timely harvesting and pruning of overgrown trees, could further minimize the likelihood of coconut falls. Urban planning measures that prioritize the placement of coconut trees away from high-traffic areas or densely populated zones should also be considered.

Policy measures could support these efforts by promoting the adoption of safer coconut tree agronomy practices. For example, encouraging the cultivation of hybrid dwarf coconut varieties, which are shorter and easier to manage, could reduce both occupational and incidental risks. Government-backed programs, such as Kera Suraksha Insurance Scheme in India for coconut workers, could provide financial support for medical expenses related to coconut tree-related injuries, alleviating the out-of-pocket expenditures on affected families.

While this study provides valuable insights, certain limitations must be acknowledged. As a retrospective analysis, it relies on existing medical records, which may contain incomplete or inaccurate data. Additionally, the focus on a single tertiary care centre in coastal India limits the generalizability of the findings to other regions with different environmental and cultural contexts. Mild injuries treated outside the hospital or unreported cases were likely excluded, potentially underestimating the true burden of coconut-related trauma.

Future research should address these limitations by adopting a prospective design to capture a more comprehensive dataset, including injuries managed in community settings. Expanding the study to encompass multiple geographic regions would provide a more representative understanding of injury patterns and risk factors. Community-based surveys to assess public awareness of coconut tree-related risks could guide the development of targeted interventions. Pilot studies exploring the feasibility and effectiveness of preventive measures, such as safety nets and hybrid tree varieties, would also be valuable in informing policy recommendations.

## Conclusions

Coconut fall-related injuries, though often overlooked, pose a significant public health challenge in tropical regions, particularly among middle-aged adults and individuals exposed to occupational hazards like tree climbing and farming. Our findings reveal that these injuries range from mild soft tissue injuries to severe, life-threatening conditions, such as traumatic brain injuries and thoracic trauma, with increased incidence during the post-monsoon period. While no fatalities were reported in this study, the documented injuries underline the serious health implications of falling coconuts.

The results emphasize the urgent need for targeted preventive strategies, including regular pruning of coconut trees, installation of safety nets, and community education campaigns aimed at high-risk groups like outdoor workers and residents near coconut trees. Future efforts should focus on implementing evidence-based safety interventions and expanding research to include broader geographic and demographic data. Implementing these strategies can help reduce the burden of such injuries while safeguarding the livelihoods tied to coconut trees.

## Electronic supplementary material

Below is the link to the electronic supplementary material.

Supplementary Material 1

## Data Availability

No datasets were generated or analysed during the current study.
